# Very advanced maternal age and morbidity in Victoria, Australia: a population based study

**DOI:** 10.1186/1471-2393-13-80

**Published:** 2013-03-27

**Authors:** Mary C Carolan, Mary-Ann Davey, Maryanne Biro, Michelle Kealy

**Affiliations:** 1 School of Nursing and Midwifery, Victoria University, St Alban’s Campus, PO Box 14228, Melbourne 8001, Australia; 2 Consultative Council on Obstetric and Paediatric Mortality and Morbidity, Clinical Councils Unit, Department of Health, 50 Lonsdale Street, Melbourne, Victoria 3000, Australia; 3 Mother and Child Health Research, La Trobe University, Melbourne, Australia; 4 School of Nursing and Midwifery, Monash University, Clayton Campus, Wellington Road, Clayton 3800, Australia

**Keywords:** Perinatal death, Very advanced maternal age, Low birth weight, Preterm birth, Haemorrhage

## Abstract

**Background:**

In Australia, approximately 0.1% of births occur to women 45 years or older and this rate has been increasing in recent years. There are however, few population based studies examining perinatal outcomes among this age group. The aim of this study was to determine the maternal and perinatal outcomes of pregnancies in women aged 45 years or older compared to women aged 30–34 years.

**Methods:**

Data on births at 20 or more weeks’ gestation were obtained from the Victorian Perinatal Data Collection for the years 2005 and 2006. We examined selected maternal and perinatal outcomes for women of very advanced maternal age (VAMA) aged 45 years or older (n = 217) and compared them to women aged 30–34 years (n = 48,909). Data were summarised using numbers and percentages. Categorical data were analysed by Chi-square tests and Fisher’s exact test. Comparisons are presented using unadjusted odds ratios, 95 percent confidence intervals (CIs) and p-values.

**Results:**

Women aged 45 years and older had higher odds of gestational diabetes (OR 2.05; 95% CI 1.3–3.3); antepartum haemorrhage (OR 1.89; 95% CI 1.01–3.5), and placenta praevia (OR 4.88; 95% CI 2.4–9.5). The older age-group also had higher odds of preterm birth between 32–36 weeks (OR 2.61; 95% CI 1.8–3.8); low birth-weight (<2,500 gr) (OR 2.22; 95% CI 1.5–3.3) and small for gestational age (OR 1.53; 95% CI 1.0–2.3). Stratified analysis revealed that VAMA was most strongly associated with caesarean section in primiparous women (OR 8.24; 95% CI 4.5, 15.4) and those using ART (OR 5.75; 95% CI 2.5, 13.3), but the relationship persisted regardless of parity, ART use and plurality. Low birthweight was associated with VAMA only in first births (OR 3.90; 95% CI 2.3, 6.6), while preterm birth was more common in older women for both first (OR 3.13; 95% CI 1.8, 5.3) and subsequent (OR 2.08; 95% CI 1.2, 3.5) births, and for those having singleton births (OR 2.11; 95% CI 1.3, 3.4), and those who did not use ART (OR 2.10; 95% CI 1.3, 3.4). Preterm birth was very common in multiple births and following ART use, regardless of maternal age.

**Conclusions:**

This study demonstrates that women aged 45 years and older, in Victoria, Australia, have higher rates of pregnancy and perinatal complications, compared to women aged 30–34 years.

## Background

In Australia and most high-income countries, childbearing over 35 years has become increasingly common over the past 30 years [1-3]. This increasing trend is well established for women aged 35–44 years [1-3], however, more recently, a new trend in older childbearing has emerged, and increased rates of pregnancy are now seen among women aged 45 years or older [4-6]. This new trend is driven, in part, by developments in assisted reproductive technologies (ART), such as oocyte donation. These new technologies offer childbearing options for women in the 5th and 6th decades of life [5,7,8]. At present, approximately 0.1–0.2% births in Australia are to women aged 45 years or older [1] and this trend is likely to continue as newer technologies emerge. However, despite this growing interest in childbearing at the extremes of advanced maternal age, to date, most studies have evaluated pregnancy outcomes in women of advanced maternal age 35–44 years, and currently, there is limited evidence to guide health professionals engaged in the care of pregnant women of very advanced maternal age.

Although the evidence is limited, pregnancy after 45 years has been associated with an increased risk of adverse maternal and perinatal outcomes, such as hypertensive disorders, gestational diabetes, caesarean birth, [6,9-11], placenta praevia [11], postpartum haemorrhage [10], low birth-weight (LBW)[11], preterm birth [11], and stillbirth [12,13]. Current evidence also suggests that adverse pregnancy outcomes among women aged 45 years or older are in excess of those experienced by women of less advanced maternal age (35–39 years) [8,13,14]. However, despite this pattern of increased risk, some questions remain unanswered. For example, the impact of factors such as primiparity and pre-existing maternal health, on maternal and perinatal outcomes, is not well understood. Moreover, the socio-demographics of very advanced maternal age have also changed in the last 2 decades, and this factor may influence contemporary maternal and perinatal outcomes. Currently, pregnant women aged 45 years or older are more likely to be of low parity and higher socio economic circumstance [6,15], and a sizeable percentage may also use ART to conceive [4,8,16]. This is in contrast to previously, when very advanced maternal age was more often associated with high parity and low socio economic status [9,10,17,18], and may have represented the final birth for women, who for social or cultural reasons, continued to childbear until menopause [9,19]. These changing social circumstances may impact positively on pregnancy outcomes, as higher socio economic circumstance is associated with more favourable pregnancy outcomes and fewer stillbirths [17,20,21].

Most previous studies of very advanced maternal age are hospital based studies, and to date very few population based studies have investigated outcomes among women aged 45 years or older. The aim of this state-wide population-based study was to describe the incidence of a range of maternal and perinatal outcomes for women aged 45 years or older, compared to women aged 30–34 years. An earlier study has reported on findings among women aged more than 35 years [22].

## Methods

Data on all births in Victoria, Australia, over 20 weeks’ gestation, for the years 2005 and 2006, were obtained from the Victorian Perinatal Data Collection (VPDC). In the VPDC, gestation is reported in completed weeks and twenty weeks’ gestation marks the cutoff point between miscarriage and fetal loss [23].

VPDC Information is collected during pregnancy and at the birth and includes demographic data, reproductive history, pre-existing conditions and complications during pregnancy, birth, and the neonatal period. Most often, data collection is performed by the midwife attending the birth. For this study, very advanced maternal age was defined as 45 years or older, and was calculated according to maternal age when the women gave birth. Data were analysed for women aged 45 years or older compared with women aged 30 to 34 years, the median age for giving birth in Victoria, Australia. Terminations of pregnancy were excluded from the analysis. Maternal medical conditions were confined to pre-existing hypertension and pre-existing diabetes. Complications of pregnancy included pre-eclampsia, gestational diabetes, antepartum haemorrhage, placenta previa, post partum haemorrhage (blood loss >500 mL in the 24 hours following vaginal birth, or >750 mL following caesarean section), and multiple birth. Method of birth was categorised as caesarean section (elective or emergency)/no caesarean section. The use of any assisted reproductive technology was reported to VPDC as assisted conception.

Perinatal outcomes included: pre-term birth, categorised as birth at 32–36 weeks’ gestation and very pre-term birth at ≤ 31 weeks’ gestation; extremes of birth-weight, including low birth-weight, (< 2,500 g) and macrosomia, (> = 4,500 g); and small for gestational age (SGA) (< 10th centile birth-weight, for gestation, sex and plurality [24,25]. Perinatal deaths were assessed using the Consultative Council on Obstetric and Paediatric Mortality and Morbidity (CCOPMM), definition of perinatal death, as comprising all fetal deaths (of at least 20 weeks gestation or 400 grams birth-weight if gestation is unknown), together with all neonatal deaths (all live born babies who died within 28 completed days of birth)[26]. The study was approved by the Human Ethics Research Committee, Department of Health Victoria, Human Ethics Research Committee Victoria University, and CCOPMM.

### Statistical analysis

Frequencies were used to describe the characteristics of women aged 45 years or older, such as pre-existing medical conditions, parity and use of ART. Proportions and chi-square tests were used to explore the relationships between maternal age and a number of other variables, including maternal outcomes such as pre-eclampsia, gestational diabetes and haemorrhage; and perinatal outcomes such as birth-weight, preterm birth and SGA. Odds ratios and their 95% confidence intervals (95% CI) were calculated for the main outcome measures. Analyses were carried out using SPSS version 19 [27]. Fishers exact p-values were reported when cell sizes were less than 5.

## Results

In Victoria, Australia, 133,359 women gave birth during 2005 and 2006. Of these women, 36.7% were aged 30–34 years. A further 0.16% were aged 45 years or older, including 5 women who were aged more than 50 years (Table 1).

**Table 1:** Confinements in Victoria 2005–2006 by maternal age

**Maternal age at confinement**	**n**	**%**
19 years and younger	3,683	2.8
20–24 yrs	15,116	11.3
25–29 yrs	33,630	25.2
**30–34 yrs**	**48,909**	**36.7**
35–39 yrs	26,931	20.2
40–44 yrs	4,871	3.7
**45–50 yrs**	**212**	**0.16**
**>50 years**	**5**	**0.0**
Unknown age	2	0.0
**Total**	**133,359**	**100.0**

Table 2 summarises maternal characteristics and pre-existing conditions. Almost two thirds of women in both age groups were multiparous. Women aged 45 years or older were more likely to have pre-existing hypertension, but there was only weak evidence of an increased risk of pre-existing diabetes. Women in the older group were 12 times more likely to have used assisted reproductive technologies.

**Table 2:** Maternal characteristics

	**30–34 years**		**45+ years**				
	**n**	**%**	**n**	**%**	**Odds ratio**	**95% CI**	**p-value**
**Pre existing Hypertension**	558	1.1%	6	2.8%	2.46	(0.98, 5.8)	0.04
**Pre existing Diabetes**	256	0.5%	<5	1.4%	2.66	(0.7, 8.7)	0.108
**Parity**							
**Primip**	19247	39.4%	76	35.0%			0.193
**Multip**	29662	60.6%	141	65.0%			
**Assisted conception**	1346	2.8%	54	24.9%	11.71	(8.5, 16.2)	<0.001

Complications of pregnancy are shown in Table 3. Older women were at increased odds of gestational diabetes (OR 2.05; 95% CI 1.3–3.3), antepartum haemorrhage (OR 1.89; 95% CI 1.01–3.5) and placenta praevia (OR 4.88; 95% CI 2.4–9.5). However, there was weak evidence for an increased risk of pre-eclampsia. Women aged 45 years or older were also more likely to have at least one of these four conditions (OR 2.16; 95% CI 1.52, 3.05) and were more likely to have a multiple birth (OR 3.16; 95% CI 1.7–5.7) and caesarean section (OR 2.62; 95% CI 2.0–3.5). They were no more likely to have had a postpartum haemorrhage.

**Table 3:** Complications of pregnancy

	**30–34 yrs**		**45+ yrs**				
	**n**	**%**	**n**	**%**	**OR**	**95% CI**	**p-value**
**Pre-eclampsia**	1237	2.5%	10	4.6%	1.86	(0.9, 3.6)	0.052
**Gestational diabetes**	2425	5.0%	21	9.7%	2.05	(1.3, 3.3)	0.0014
**Antepartum haemorrhage**	1466	3.0%	12	5.5%	1.89	(1.01, 3.5)	0.0293
**Placenta praevia**	479	1.0%	10	4.6%	4.88	(2.4, 9.5)	<0.001
**At least one of the above complications**	5030	10.3	43	19.8	2.16	(1.5, 3.1)	<0.001
**None of the above complications**	43879	89.7	174	80.2			
**Multiple birth**	983	2.0%	13	6%	3.11	(1.7, 5.6)	<0.001
**Postpartum haemorrhage**	4641	9.5%	21	9.7%	1.02	(0.6, 1.6)	0.9247
**Caesarean section**	15,683	32.1%	120	55.3%	2.62	(2.0, 3.5)	<0.001

Perinatal outcomes are shown in Table 4. Babies born to older women were at higher odds of preterm birth between 32–36 weeks’ gestation (OR 2.61; CI 95%, 1.8–3.8 ); low birth-weight < 2,500 g (OR 2.22; CI 95%, 1.5–3.3) and small for gestational age (OR 1.53; CI 95%, 1.0–2.3). The evidence for an increased risk of birth before 32 weeks was weak, and older women were no more likely than younger women to have a macrosomic baby. There were too few perinatal deaths for meaningful comparison (data not reported).

**Table 4:** Perinatal outcomes

	**30–34 yrs**		**45+ yrs**				
	**n**	**%**	**n**	**%**	**OR**	**95% CI**	**P value**
**Gestation**							
< 31 weeks’	655	1.4%	5	2.2%	1.84	(0.7, 4.6)	0.2065
32–36 weeks’	3041	6.1%	33	14.3%	2.61	(1.8, 3.8)	<0.001
**Birth-weight**							
<2,500 g	3042	6.1%	29	12.6%	2.22	(1.5, 3.3)	<0.001
> = 4,500 g	948	1.9%	<5	1.7%	0.98	(0.3, 2.7)	1
**Small for gestational age**	3861	7.7%	26	11.3%	1.53	(1.0, 2.3)	0.0404
**Perinatal death**		6.9/1000 births		13.0/1000 births			

There is strong evidence that women aged 45 years or older were more likely to have a caesarean section regardless of parity, plurality and whether or not they used ART. The odds were increased 8-fold for first births at 45 years or older compared with first births to younger women, and 6-fold for those who used ART at 45 years or older compared with younger women who used ART.

There is also strong evidence that women aged 45 years or older were more likely than younger women to have a preterm birth regardless of parity. The strong effect of maternal age on PTB was limited to singleton births and those that did not result from ART. Rates of PTB were similar and very high in both age groups for multiple births and those who used ART. After stratification by parity, plurality and use of ART, the relationship between VAMA and low birthweight remained strong only for first births (Table 5).

**Table 5:** Caesarean section, preterm birth and low birthweight by parity, ART and plurality

	**30–34 years**		**45+ years**		**OR**	**95% CI**	**p-value**
	**n**	**%**	**n**	**%**			
CS							
primip	6,729	35.0	62	81.6	8.24	(4.5, 15.4)	<0.0001
Multi	8,954	30.2	58	41.1	1.62	(1.1, 2.3)	0.0047
ART	673	50.0	46	85.2	5.75	(2.5, 13.3)	<0.0001
No ART	15,010	31.6	74	45.4	1.80	(1.3, 2.5)	0.0001
Singleton	14,991	31.3	107	52.5	2.42	(1.8, 3.2)	<0.0001
Multiple	692	70.4	13	100.0	undefined		0.0139*
Preterm birth							
Primip	1,766	9.0	20	23.5	3.13	(1.8, 5.3)	<0.0001
Multi	1,930	6.4	18	12.4	2.08	(1.2, 3.5)	0.0032
ART	423	26.3	16	25.0	0.93	(0.5, 1.7)	0.8114
No ART	3,273	6.8	22	13.2	2.10	(1.3, 3.4)	0.0009
Singleton	2,594	5.4	22	10.8	2.11	(1.3, 3.4)	0.0007
Multiple	1,102	55.7	16	61.5	1.27	(0.5, 3.0)	0.5486
Low Birthweight							
Primip	1,529	7.8	21	24.7	3.90	(2.3, 6.6)	<0.0001
Multi	1,513	5.0	8	5.5	1.11	(0.5, 2.3)	0.7808
ART	357	22.2	15	23.4	1.07	(0.6, 2.0)	0.8218
No ART	2,685	5.6	14	8.4	1.56	(0.9, 2.8)	0.1070
Singleton	2,038	4.3	12	5.9	1.41	(0.8, 2.6)	0.2501
Multiple	1,004	50.8	17	65.4	1.83	(0.8, 4.5)	0.1397

*Fisher’s exact test.

## Discussion

Having a baby when aged 45 years or older remains uncommon in Australia, at 0.1–0.2% of all births [28]. In this study, 0.16% of women giving birth in Victoria, during the study period, were in this age group. However, rates of very advanced maternal age are increasing in Australia and globally and are likely to continue to increase as more sophisticated reproductive technologies become available [4]. These factors make it an important area of future study. The principal aim of this study was to determine maternal and perinatal outcomes of pregnancies in women aged 45 years or older, giving birth in Victoria, Australia, compared to women in the median age group of 30–34 years. We found that despite considerable emphasis on chronic illness in other studies, women in our study had low levels of pre-existing disease and most (72.2%) conceived naturally. These features suggest a generally healthy cohort. Nonetheless, women aged 45 years or older were at higher odds of experiencing a number of maternal and perinatal complications. These findings are discussed below.

### Pre-existing conditions

Pre-existing hypertension occurred at more than twice the rate among women aged 45 years or older, compared to women aged 30–34 years. However, there were too few older women with pre-existing diabetes to be confident of an increase in the older group. Nonetheless, recorded rates of 2.8% (hypertension) and 1.4% (diabetes) are modest compared to other studies examining pregnancy at 45 years and older [9,11,19]. Yogev et al., [11], for example, found rates of 6.8% (hypertension) and 4.5% (diabetes) among women aged more than 45 years in Israel, while Dildy et al., [9] found equivalent rates of 3.8% and 3.8% in the US, and Abu Heija et al., [19] found rates of 9.6% and 4.4% respectively in Jordan. Only one study was located that had lower rates of these two chronic conditions. Callaway et al., [6], in a study of Australian women, found very low rates, with just one woman out of 77 presenting with pre-existing hypertension and none with pre-existing diabetes. The low incidence of pre-existing disease seen in both our study and Callaway et al., [6] may relate to a tendency in Australia, for older maternal age to be associated with higher socio-economic status and generally healthy women [29,30]. Although we could not establish the degree of multiparity in our study, subjects aged 45 years or older in studies from Hong Kong, Jordan and Utah, US, tended to be of very high parity, and this factor may have contributed to the increased rates of pre-existing disease seen in those studies [9,19,31].

### Pregnancy complications

Similar to other studies in this area [8,9,11,19], very advanced maternal age in our study was characterised by an increased incidence of pregnancy complications such as pre-eclampsia, gestational diabetes, antepartum haemorrhage and caesarean section. However, although the rate for pre-eclampsia among women 45 years or older was almost double the rate for younger women, at 4.6%, it was significantly lower than several comparable studies which reported rates of 10–12% [6,9-11,19,32] and very much lower than Glasser et al.’s study [8], which reported rates of pre-eclampsia at 18.3%. Glasser et al., [8] examined pregnancy outcomes among primiparous ART recipients in Israel and reported a number of extreme findings, most likely related to primiparity, extremely advanced maternal age (45–65 years), and the unscreened nature of the sample. In contrast to Glasser et al., [8], other studies that examined pregnancy morbidity among women receiving ART, generally reported on women who had been screened for pre-existing disease prior to acceptance into fertility programs [16,33]. Moreover, higher rates of pre-existing hypertension found among other study populations aged 45 years or older [9,11,19] may have influenced subsequent higher rates of pre-eclampsia, as this link is well established [34]. Gestational diabetes mellitus (GDM) rates were high in our study group, at 9.7% compared to the Australian national rate of 4.6% [35], but lower than Australia-wide rates of 13% for women aged 45–49 years [35]. Our findings are also consistent with other studies of very advanced maternal age where GDM presents at rates of 8–12% [6,9]. In contrast to these more general findings, Yogev et al., [11] and Glasser et al., [8] each reported extremely high rates of GDM at 17% and 42.7% respectively. Both these Israeli studies reported significantly higher rates of a number of outcome variables which may in part be explained by the extreme age of participants [8] and high rates of ART, which have been previously been linked to poorer pregnancy outcomes [36].

Antepartum haemorrhage was reported in our study at 5.5%, however, comparison with other studies was limited as only one other study was found to have examined this variable. Abu Heija et al., [19] found rates of antepartum haemorrhage of 10.5% in Jordan, which likely relates to trends of very high parity and continued childbearing until menopause, reported in that study. Compared to women aged 30–34 years, placenta praevia (4.6%) was more than four times as common in our study population and similar rates were found in other studies at 4.4–5.6% [11,19]. Rates for postpartum haemorrhage (PPH) were unaffected by maternal age 45 years or older (9.7% vs 9.5%) in our study, although rates for both age groups were higher than comparable studies which reported rates of 4.0%–6.35% [11,32]. It is not immediately clear why this disparity exists although differences in estimation and reporting of PPH may contribute [37]. It may also relate to the year of study as rates of postpartum haemorrhage are increasing over time [38].

Caesarean rates of 55.3% are reported in our study and this is consistent with caesarean rates in Australia overall where 45–57% of women aged 40 years and over will have a caesarean section [39,40]. Elsewhere, caesarean section for women aged 45 years or older showed little consensus. At the lower end of the scale, Dildy et al., [9], and Dulitzki et al., [10] reported rates of 31% to 40% while Yogev et al., [11] and Glasser et al., [8] reported much higher rates of 78.5% and 93.9% respectively. The lower rates reported by Dildy et al., [10] and Dulitzki et al., [11] may relate to the age of these publications which report on data from 1985–1994 and 1996 respectively. Caesarean section rates have increased significantly since this era and women in both these studies were predominantly multiparous, a feature which is also associated with lesser rates of caesarean section. In contrast, high rates of caesarean section were reported by Yogev et al., [11] (78.5%) and Glasser et al., [8] (93.9%) and may reflect increasing trends of caesarean section overall, higher numbers of primiparous women in these studies [8,11], very extreme maternal age [8] and high use of ART. Numbers of women with a previous caesarean section may also have contributed in Yogev et al’s study [11], as previous caesarean is a strong predictor for repeat caesarean [40].

### Perinatal outcomes

Adverse perinatal outcomes such as preterm birth at 32–36 weeks, low birth-weight, and birth-weight below the 10th centile were all increased 1.5–2 fold for women aged 45 years or more in this study and this increase was most obvious among primiparous women, although preterm birth was also more common in older multiparous women. These findings are consistent with trends of poorer perinatal outcomes reported in other studies [6,9,11,19,32]. However, direct comparison of specific variables was not always possible due to different study approaches and categorisation of variables. For pre-term births, there was a tendency in the literature, to report all preterm births as births at < 37 weeks gestation and therefore our total rate of 17.5% (32–36 weeks and before 32 weeks) is presented here for comparison. Our study rates were less than rates of 18.3%–29.8% reported by Dulitzki et al., [10], Glasser et al., [8] and Yogev et al., [11] and slightly higher than rates of 11.6–15.2% found by Callaway et al., [6] and Dildy et al., [9]. Only one study, Yogev et al., [11] reported on pre-term birth at less than 32 weeks gestation and found rates of 2.3% which are very similar to our rates of 2.2%. For low birth-weight, our rates of 12.6% are consistent with Callaway et al’s [6] and Dulitzki et al’s [10] findings of 10–11% and lower than rates reported by Dildy et al., [9] and Glasser et al., [8] at 17.3%–27.8%. Rates of macrosomia, on the other hand, were very similar among our study population, compared to the comparison group, but rates overall were very low. Comparison with the literature was hampered by the tendency, in other studies, to consider macrosomia at 4.0 kg compared to 4.5 kg in our study [9,10].

Small for gestational age was the most common weight measurement reported by other studies of women aged 45 years or older [6,8,11,32,41,42] and rates ranged from 4.8% to 30%. Our rates of 13.8% are slightly higher that the lower range reported in this literature, and 1.5–2 fold higher than the 5–9% rate found in the general Australian population [39]. At the lower end of the scale, Shrim et al., [32], Jacobsson et al., [41], Callaway et al., [6], and Yogev et al., [11], reported SGA rates of 4.8%–11.3%. At the higher end of the scale, Israeli studies by Glasser et al., [8] and Simchen et al., [42] reported rates of 25% and 30% respectively. The current study found increased rates of PTB for older women having first births as well as subsequent births, for singleton but not multiple births, and for those who did not use ART but not for those who did use it. As would be expected, rates of PTB were substantially higher for multiple rather than singleton births, and for those who used ART regadless of maternal age.This association between preterm birth, very advanced maternal age and primiparity is found in other studies [8,15,43] as is the link with SGA [8,11]. Cano et al., [44] suggest that SGA in this maternal age group is likely related to poorer placental development and generally poorer performance of the aging uterus. We found that LBW, but not SGA was associated with multiple birth in our population and this is in contrast with the literature generally, where older maternal age is more commonly associated with higher infant birthweight among multiple pregnancies [45-51]. We also found that babies born after ART and multiples had higher rates of low birthweight regardless of maternal age, but VAMA increased the risk of PTB only for first births.

There is however some difficulty with direct comparison, as very few studies examined multiple pregnancy in women of very advanced maternal age. Most considered advanced maternal age in a single group, ≥35 years [46,47,49], or in two groups ≥35 and ≥40 years [48,51]. Studies explicitly examining outcomes among women ≥ 45 years typically report on very small numbers of women with multiple births and do not analyse outcomes separately for singletons and multiples [6,42]. In general, women of very advanced maternal age are considerably more likely to have multiple births, and in this study, multiple birth rates were approximately 6% for the study group, which is three fold the rate reported among younger women. This rate is also considerably in excess of rates for multiple births occurring in the gereral population, in Australia of approximately 2.5% to 3% [39].

The absolute number of perinatal deaths to women of 45 years or older was very low in this study, and therefore valid comparison with younger women was not possible. Rates are also subject to random fluctuation from year to year [42] and for this reason, continued monitoring is recommended to determine if a pattern of age related increase in perinatal death is occurring. Our low rates of perinatal death are similar to recorded rates of 0–1% [6,9] and considerably less than findings in Yogev et al.’s [11] study of 7.4%. However, direct comparison with other studies was difficult as infant death was frequently reported in other studies as stillbirth [13] or neonatal death (0–28 days) alone [19,41], while we used perinatal deaths (stillbirths and neonatal deaths). This combined approach was used because perinatal death is a more meaningful outcome than stillbirth or neonatal death alone, and because of the small number of deaths to women in the older age group.

### Outcomes were generally favourable

Overall, there was evidence, in this study, of increased rates of pre-existing chronic conditions such as hypertension and diabetes; higher rates of pregnancy complications, such as pre-eclampsia, gestational diabetes and placenta praevia; and; higher rates of perinatal complications such as preterm birth, low birth-weight and SGA. These findings have ramifications for women and their families, and for clinicians and health services providing maternity care. They foreshadow an increasing demand on maternity services and resources as trends of very advanced maternal age continue. Nonetheless, this study also provides some reassuring findings and maternal and perinatal outcomes were favourable for the vast majority of women and babies. Moreover, the absolute rate of perinatal death remains low, at generally less than 10 per thousand births in high income countries such as Australia [12]. This suggests that with careful prenatal care, most women in this age group will achieve a live birth. This information may assist with risk counselling for contemporary mothers of very advanced maternal age.

Potential areas for future study include the investigation of long-term outcomes for infants born to women of this age group, as high rates of preterm birth, LBW and SGA may impact on the future health of this group of children.

### Strengths and limitations

This large population study provides useful information about perinatal outcomes for women aged 45 years and over in Victoria, Australia. The size and established accuracy of the state-wide population database used, is also a strength in this study [52], as is the ability to stratify into various maternal age groups. The study does however, have limitations and there are few women in this age group, which makes comparison with other age groups difficult. The dataset also did not include socio-demographic details such as income and educational level, or smoking and these variables may have impacted on outcomes among women of very advanced maternal age. Additionally, the dataset did not include data on body mass index [BMI], and rates of obesity may have impacted negatively on pregnancy outcomes among this group. Finally, the use of ART is likely under-reported in all age groups and this factor may also impact on findings.

## Conclusion

This study demonstrates that women aged 45 years and older have higher rates of pregnancy and perinatal complications, compared to women aged 30–34 years. However, although pregnancy of very advanced maternal age was associated with greater levels of maternal complications, outcomes were generally favourable. This feature may relate to the general health of women in our study.

## Abbreviations

ART: Assisted reproductive technologies; VPDC: Victorian perinatal data collection; GDM: Gestational diabetes mellitus; GDM: Small for gestational age; CCOPMM: Consultative council on obstetric and paediatric mortality and morbidity; PPH: Postpartum haemorrhage; LBW: Low birth weight.

## Competing interests

There were no competing interests.

## Authors’ contributions

MC conceived the study, and participated in the study design and data analysis and drafted the manuscript. MAD contributed in terms of data extraction and statistical analysis of the data, and contributed intellectually to the design of the project and editing of the manuscript. MAB contributed intellectually to the design of the project, data analysis and editing of the manuscript. MK contributed intellectually to the design of the project and critically reviewed the text. All authors read and approved the final manuscript.

## Pre-publication history

The pre-publication history for this paper can be accessed here:

http://www.biomedcentral.com/1471-2393/13/80/prepub
